# EHA Endorsement of ESMO Clinical Practice Guidelines for Marginal Zone Lymphomas

**DOI:** 10.1097/HS9.0000000000000351

**Published:** 2020-03-11

**Authors:** Marco Ladetto, Martin Dreyling

**Affiliations:** 1 Division of Hematology, Azienda Ospedaliera SS. Antonio e Biagio e Cesare Arrigo, Alessandria, Italy; 2 Department of Medicine III, University Hospital, LMU, Munich, Germany.

EHA and ESMO recently agreed to collaborate in the production of European Guidelines for different hematological malignancies. As a first step, a number of completed guidelines will be reviewed by the corresponding EHA Scientific Working Groups in a standardized review process. The ESMO guideline on Marginal Zone Lymphomas^1^ represents the first example of this collaboration and has been endorsed by the EHA LyG (www.ehalyg.org).

The definition of marginal zone lymphoma (MZL) encompasses a number of different entities including extra nodal MZL (EMZL) of mucosa-associated lymphoid tissue (MALT), also known as MALT lymphoma, splenic MZL (SMZL) with or without villous lymphocytes and nodal MZL (NMZL) with or without monocytoid B cells. These entities and particularly MALT lymphoma are far from being homogeneous in terms of etiology, biology, clinical presentation, and treatment. Increasing knowledge has been accumulated on the biology and pathogenesis of several of these entities, and histological characterization appears straightforward compared to the past. Due to rarity and heterogeneity of this subgroup, very few randomized phase III clinical trials have focused on MZL. As a consequence, most of the evidence is still arising from retrospective or some clinical prospective experiences, as well as from studies enrolling multiple histological subtypes of indolent lymphomas. Nevertheless, substantial progress has been made over recent years including results from few well-conducted histotype-specific and (for EMZL) site-specific prospective clinical trials. The combination of rituximab and chlorambucil in EMZL or the benefit of ibrutinib in relapsed MZL are 2 prominent examples of recent practice-changing advances in the field.

It should be noted that, until now, European guidelines have not been available for most MZL-related conditions. ESMO guidelines were limited to gastric EMZL and only few and often obsolete national guidelines have become available. The current MZL clinical practice guidelines^1^ are therefore an ambitious and unprecedented effort, as all MZL entities have been covered in detail. This is particularly relevant for SMZL where considerable heterogeneity among treatment choices exist ranging from splenectomy, antiviral treatment, immunotherapy and immunochemotherapy.

The purpose of any guideline is to provide the best available clinical guidance in an easy and accessible way that could be effectively translated in the everyday practice. It must be realized that the field still lacks high levels of evidence in some critical points that impact the management of our patients. Nevertheless, hematologists, clinical oncologists, radiation oncologists, biologists and pathologists who work on the development of these guidelines have struggled to generate a balanced and effective tool that hopefully will be of substantial help for clinicians dealing with MZL in Europe and beyond.
